# Amyloid β accelerates age-related proteome-wide protein insolubility

**DOI:** 10.1007/s11357-024-01169-1

**Published:** 2024-05-16

**Authors:** Edward Anderton, Manish Chamoli, Dipa Bhaumik, Christina D. King, Xueshu Xie, Anna Foulger, Julie K. Andersen, Birgit Schilling, Gordon J. Lithgow

**Affiliations:** 1 The Buck Institute for Research On Aging, 8001 Redwood Blvd, Novato, CA 94945 USA; 2 USC Leonard Davis School of Gerontology, University of Southern California, 3715 McClintock Ave, Los Angeles, CA 90191 USA

**Keywords:** Amyloid β, Proteostasis, Protein insolubility

## Abstract

Loss of proteostasis is a highly conserved feature of aging across model organisms and results in the accumulation of insoluble protein aggregates. Protein insolubility is also a unifying feature of major age-related neurodegenerative diseases, including Alzheimer's Disease (AD), in which hundreds of insoluble proteins associate with aggregated amyloid beta (Aβ) in senile plaques. Despite the connection between aging and AD risk, therapeutic approaches to date have overlooked aging-driven generalized protein insolubility as a contributing factor. However, proteins that become insoluble during aging in model organisms are capable of accelerating Aβ aggregation in vitro and lifespan in vivo. Here, using an unbiased proteomics approach, we questioned the relationship between Aβ and age-related protein insolubility. Specifically, we uncovered that Aβ expression drives proteome-wide protein insolubility in *C. elegans*, even in young animals, and this insoluble proteome is highly similar to the insoluble proteome driven by normal aging, this vulnerable sub-proteome we term the core insoluble proteome (CIP). We show that the CIP is enriched with proteins that modify Aβ toxicity in vivo, suggesting the possibility of a vicious feedforward cycle in the context of AD. Importantly, using human genome-wide association studies (GWAS), we show that the CIP is replete with biological processes implicated not only in neurodegenerative diseases but also across a broad array of chronic, age-related diseases (CARDs). This provides suggestive evidence that age-related loss of proteostasis could play a role in general CARD risk. Finally, we show that the geroprotective, gut-derived metabolite, Urolithin A, relieves Aβ toxicity, supporting its use in clinical trials for dementia and age-related diseases.

**Supplementary Information:**

The online version contains supplementary material available at 10.1007/s11357-024-01169-1.

## Introduction

Insoluble protein aggregates accumulate during normal aging across eukaryote species, from yeast to mice [1–9]. In *Caenorhabditis elegans*, insoluble protein accumulation is strongly associated with aging and compounds that slow the accumulation of insoluble proteins extend lifespan [4, 10]. In humans, protein insolubility is a unifying feature of all major, age-related neurodegenerative diseases, despite the canonical protein associated with aggregation being different in each disease. In AD, genetic, biochemical, and animal model data support a central role for Aβ, and in particular the Aβ_1-42_ peptide, in the formation of aggregates and neurodegeneration [11–20]. Despite this, the causal role of Aβ in AD is unclear. Aggregates isolated from AD patient brains contain hundreds of insoluble proteins in addition to Aβ and their orthologues have been shown to aggregate during normal aging in *C. elegans *[4, 5, 9, 21, 22]. While aging remains the single greatest risk factor for AD, therapeutic approaches have failed to consider age-related, generalized protein insolubility as a contributing factor instead focusing on Aβ and Tau [23–26]. Data suggest there may be a causal role for the insoluble proteome in AD pathogenesis; for example, insoluble protein extracts from old but not young animals, accelerate the aggregation of Aβ in vitro [27]. We questioned if the reverse was also true: can Aβ drive the insolubility of proteins that tend to aggregate during aging? Could age-dependent insoluble proteins interact with Aβ in a destructive feedforward cycle in the aged brain, leading to an acceleration of protein insolubility in AD? We propose that identifying which proteins are affected by Aβ in this way would uncover novel mechanisms to prevent the cytotoxic effect of Aβ.

Here, using an unbiased proteome-wide approach, we tested the effect of Aβ on protein insolubility in a well-established *C. elegans* model of Aβ toxicity [28]. We demonstrate that Aβ expression drives a dramatic increase in proteome-wide protein insolubility even in young adults, but that this insoluble proteome is highly similar to the insoluble proteome that occurs during normal aging. This vulnerable insoluble sub-proteome we term the core insoluble proteome (CIP). We find that the CIP is highly enriched with neurodegenerative disease (ND) associated proteins and modifiers of ND pathology across AD, Parkinson’s, and Huntington’s disease models.

By analyzing publicly available human GWAS data, we found the CIP to be replete with biological processes implicated in not only NDs but a wide array of chronic, age-related diseases, suggesting that age-related protein insolubility may play a role in more than just neurodegeneration. Finally, we show that by targeting the CIP both genetically and pharmacologically we could uncover candidates that modify Aβ toxicity in vivo. Taken together, our findings provide insights into the link between Aβ and age-related protein insolubility and suggest a role for generalized insolubility in NDs and chronic, age-related diseases more broadly.

## Results

### Aβ expression drives proteome-wide protein insolubility

**Fig. 1:**
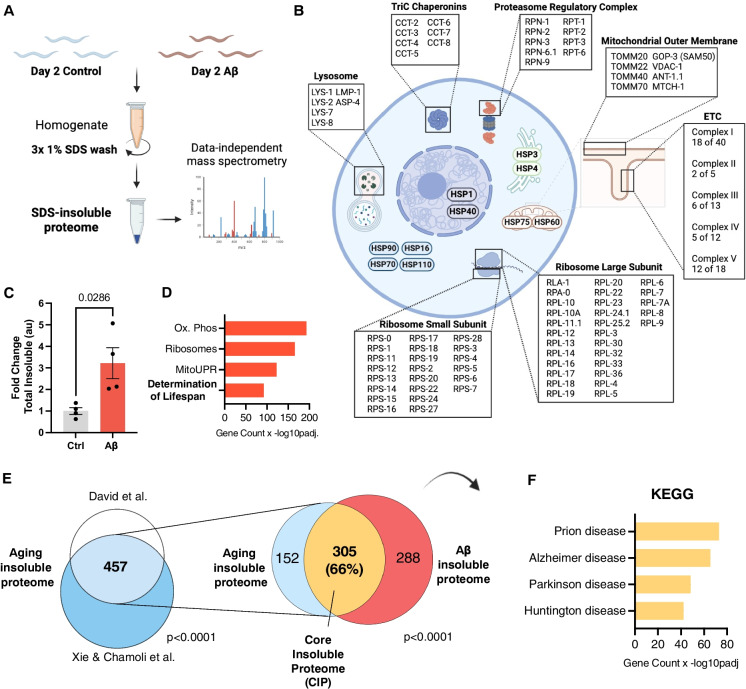
Aβ drives proteome-wide protein insolubility that resembles normal aging. **A** Schematic of experimental procedure, created with Biorender.com. **B** Schematic overview of proteostasis machineries and mitochondrial proteins enriched in the Aβ-driven insoluble proteome. **C** Total insoluble protein Intensity in worms expressing Aβ vs genotype control strain CL2122, all values normalized to the average of the control in each experiment n = 4, error bars = SEM, Mann-Whitney Test, created with Biorender.com. **D** Barplot of selected enriched GO terms, KEGG pathways and Wikipathways in the Aβ insoluble proteome, Benjamini Hochberg FDR <5% (see supplementary tables for complete list). **E** Left: Overlap of proteins enriched in the insoluble proteome of old *C. elegans* from two publicly available datasets [5, 28] *p* < 0.0001 Fischer’s exact test. Right: Overlap of aging insoluble proteome with Aβ-driven insoluble proteome, *p* < 0.0001 Fischer’s exact test. **F** KEGG disease pathways enrichment of human orthologues of core insoluble proteome, Benjamini Hochberg FDR < 5%

To interrogate the effect of Aβ on proteostasis we utilized the *C. elegans* strain, GMC101, which expresses the human, pro-aggregating, and pathogenic Aβ_1-42_ peptide in muscle tissue, here referred to as simply Aβ [17, 29]. When cultured at 20 °C, animals exhibit Aβ at low levels but at 25 °C they induce elevated levels of Aβ. After 24 h of Aβ induction, > 80% paralyze [29]. We allowed animals to develop at 20 °C, i.e., without Aβ expression, and then moved them to 25 °C at the beginning of adulthood. Animals were then collected after 24 h. We extracted insoluble, aggregated proteins by serial washing lysates with 1% sodium dodecyl sulfate (SDS) buffer. We used protein mass spectrometry and data independent acquisitions (DIA) to identify and quantify proteins, as previously described [28, 30, 31] (Fig. 1A). Background genotype-control animals (CL2122) lacking the Aβ transgene were cultured and processed in parallel for comparison (Fig. 1A).

Aβ expression in young adult animals caused a robust, proteome-wide increase in the total amount of insoluble protein, impacting a wide array of essential functions (Fig. 1B, C). We identified and quantified peptides representing 1704 proteins in the insoluble fraction across four biological replicates, of which 593 proteins robustly increased due to Aβ expression across independent experiments (Fig. S1A). Cytoplasmic and organelle-specific heat shock proteins (HSPs) from across all major organelles were enriched in the insoluble fraction (Fig. S1B). Several proteins involved in maintaining proteostasis also became insoluble such as the proteasome regulatory “lid” complex, the TriC chaperonin complex, and key lysosomal proteins (Fig. 1B). Furthermore, we observed an increase in protein insolubility of almost every ribosomal subunit and translation accessory factors (Fig. 1B). Gene Ontology (GO) and pathway enrichment analysis revealed that oxidative phosphorylation (Ox. Phos), ribosomes, the mitochondrial unfolded protein response (mitoUPR), and “Determination of lifespan” were the most enriched terms in the insoluble proteome (Fig. 1D, Supp. Table 2). 42 of the 70 proteins in the *C. elegans* proteome bearing the annotation “Determination of Lifespan” became insoluble due to Aβ (Fig. 1D). Indeed, the Aβ insoluble proteome was highly enriched with mitochondrial proteins (109/593); particularly those involved in the ETC and TCA cycle (Fig. 1B, D, Supp. Table 1). Consistent with this, the mitochondrial unfolded protein response (mitoUPR) chaperone HSP-6 increased 9.2-fold in the insoluble fraction. Forty-three of the 88 ETC proteins became insoluble due Aβ expression (ETC complexes detailed in Supp. Table 3). Nuclear-encoded ETC complexes, were particularly vulnerable to insolubility (Fig. 1B) whereas mitochondrial DNA-encoded ETC complex proteins were not: only one mtDNA-encoded, complex IV protein became insoluble (Supp. Table 3). The skewed representation of nuclear-encoded subunits could be explained by deficits in the mitochondrial protein import machinery; proteins failing to locate the mitochondria could be subject to aggregation. For example, we observed a significant increase in insolubility of mitochondrial outer membrane proteins responsible for importing nuclear encoded ETC proteins. All major proteins of the TOM complex: TOM-20, TOM-22, TOM-40, and TOM-70 became insoluble (Supp. Table 1). We also identified GOP-3, the orthologue of the outer membrane complex protein SAMM50 [32], which is required for threading of mitochondrial β-barrel proteins into the outer and inner membrane bilayers [33], along with several small molecule transporters: VDAC-1, ANT-1.1 and MTCH-1 (Fig. 1B, Supp. Table 1). Interestingly, single nucleotide polymorphisms (SNPs) in several TOM complex genes have been linked with AD risk, most prominently *TOMM40*, suggesting an important role for mitochondrial protein import in the etiology of AD [34–36] (Fig. 1B).

We questioned if many of the proteins in the Aβ-driven insoluble proteome have been shown to directly interact or co-aggregate with Aβ in the literature. Comparing laser microdissection proteomics data from human AD senile plaques with the Aβ-driven insoluble proteome we found a highly significant overlap [37] (Fig. S1B, Supp. Table 4). Specifically, ~ 1/3 of the proteins which co-aggregate with Aβ in plaques also become insoluble due to Aβ expression in *C. elegans*, almost all of which are intracellular proteins (Fig. S1B & S1C, Supp. Table 4). Similarly, we find that *C. elegans* orthologues for 54% (15/28) of the proteins identified to reproducibly interact with short Aβ peptides in transfected HEK293T cells, were enriched in the insoluble proteome after Aβ expression [38] (Supp. Table 4).

### The Aβ-driven insoluble proteome is highly similar to the aging-driven insoluble proteome

Normal aging causes a significant increase in the amount of insoluble protein, and we noticed many of the same proteins that typically become insoluble during normal aging were being driven to insolubility by Aβ [4, 5, 9]. This led us to question the extent to which these two insoluble proteomes were similar. To assess this rigorously, we generated a list of proteins that robustly insolubilize during normal aging by overlapping two published aging insoluble proteomes from different laboratories [5, 28]. This resulted in an overlap of 457 proteins that reliably become insoluble during aging (*p* < 0.0001, Fischer’s exact test) (Fig. 1D). Strikingly, when we compared this with the Aβ-driven insoluble proteome, we found that 305 proteins, or 66%, became insoluble under both conditions (*p* < 0.0001, Fischer’s exact test) (Fig. 1E). Moreover, when we compared the GO biological processes (BPs) represented in the aging and Aβ insoluble proteomes, we uncovered an 89% overlap, indicating that aging and Aβ drive insolubility of proteins involved in almost identical biological processes (Fig. S1D). By comparison, previous work showed very minimal overlap (< 3%) between the proteins which become insoluble under different stress conditions, such as hsp90 inhibition, proteasome inhibition, and polyglutamine repeat expression, highlighting the remarkable similarity between the aging-driven and Aβ-driven insoluble proteomes [39]. The fact that two-thirds of proteins that become insoluble during normal aging also do so after Aβ expression in young animals suggested to us that we have identified a core set of vulnerable proteins relevant to age-related ND, which we refer to herein as the “core insoluble proteome” (CIP).

We suspected from previous work [4] that the CIP might be particularly enriched with modulators of aging and, indeed, we found that the CIP was highly enriched with modulators of lifespan. Specifically, 100 proteins, or roughly one-third of the CIP, modulate lifespan according to the GenAge database [40]; with 80% of those proteins extending lifespan when their expression is reduced or eliminated (Fig. S1E&F). Furthermore, using published whole-genome RNA interference (RNAi) screens [41–45], we found that knockdown of approximately one in six of the proteins in the CIP has been shown to improve disease pathology across Huntington’s disease (HD) and Parkinson’s disease (PD) proteinopathy models, demonstrating that the CIP is likely enriched with drivers of disease (Supp. Table 5). This led us to directly question if the orthologues of CIP proteins had been implicated in neurodegenerative proteinopathies in humans. To do so, we performed disease pathway enrichment analysis with the human orthologues of the CIP and found a highly significant enrichment for a range of neurodegenerative diseases including prion disease (Pr), AD, PD, and HD (Fig. 1F, Supp. Tables 6 and 7). In almost all cases, the proteins were annotated against all four neurodegenerative diseases, suggesting a common mechanism might underly their associations with disease (Supp. Tables 6 and 7). These data point towards protein insolubility as a common mechanism.

### The CIP is enriched with supersaturated but not intrinsically aggregation-prone proteins

**Fig. 2:**
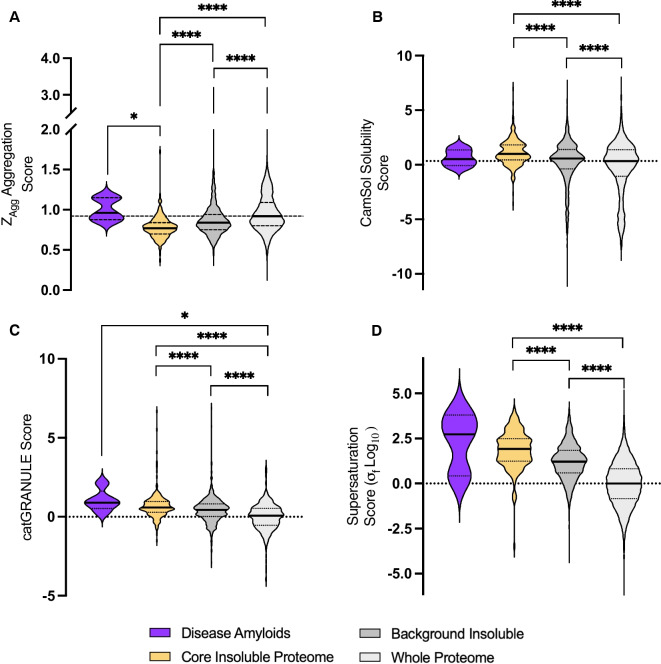
The core insoluble proteome is enriched with supersaturated but not aggregation-prone proteins. **A** Violin plot of CamSol intrinsic solubility score distribution for each proteome. **B** Violin plot of Zyggregator aggregation propensity score distribution for each proteome. **C** Violin plot of catGRANULE RNA granule prediction score for each proteome. **D** Violin plot of supersaturation (σf Log10) score distributions for each proteome. In each case, the background insoluble is all proteins identified in the insoluble proteome across any experiment and the whole proteome is the *C. elegans* reference proteome. Disease amyloids = Aβ precursor protein, ⍺-synuclein, prion protein, β-2 microglobulin, and amylin. For clarity, only comparisons that were statistically significant are shown, all other pair-wise comparisons were not significant to p.adj < 0.05, Kruskal–Wallis test with Dunn’s correction

We questioned if the intrinsic properties of the proteins of the CIP proteins might explain the common formation of insoluble aggregates under Aβ and aging. To test this, we applied a series of prediction algorithms to test if the insoluble proteome contained intrinsically aggregation-prone or insolubility-prone proteins and if the proteins in the CIP have higher liquid–liquid phase separation (LLPS) propensity than the whole proteome. We included the following five amyloidogenic, aggregation-prone proteins as “positive” comparators: Aβ precursor protein A4, prion protein, α-synuclein, β-2 microglobulin, and amylin. Surprisingly, using the Zyggregator aggregation prediction method [46], we discovered that the core insoluble proteins were significantly less aggregation-prone than the reference proteome (Fig. 2A). Similarly, using the CamSol solubility prediction score [47] we found that the CIP had a significantly higher median solubility prediction than the reference proteome (Fig. 2B). Conversely the CIP had a higher catGRANULE LLPS propensity score [48] than the reference proteome, suggesting that CIP proteins are prone to form LLPS bodies (Fig. 2C). Taken together, these predictions suggest that protein intrinsic aggregation propensity does not account for the CIPs increase in insolubility, but that LLPS propensity could play a role in their enrichment in the insoluble proteome.

Supersaturation refers to a state where the concentration of a protein in a solution exceeds its equilibrium solubility. Supersaturated proteins have been shown to be enriched in several neurodegenerative disease KEGG pathways including AD, PD, and HD [49]. Given the overrepresentation of these pathways in the CIP (Fig. 1E), we postulated that the CIP might be particularly enriched with supersaturated proteins. Indeed, we found that the average supersaturation score of the CIP was 94-fold greater than the *C. elegans* reference proteome [49] (Fig. 2D). This suggests that proteins driven to aggregate by both aging and Aβ are highly supersaturated proteins, which is consistent with previous literature showing that AD plaque proteins are particularly supersaturated [49].

This finding implies that insolubility may be simply driven by expression changes, pushing proteins beyond their solubility limit. Previous groups have shown that age-related protein insolubility does not correlate with changes in protein abundance [50]. However, to assess this in the context of the CIP, we sourced publicly available RNA sequencing data and lysate proteomics for the GMC101 Aβ model [51]. We found that 93% of CIP proteins which become insoluble due to Aβ did not change at the mRNA level and 94% did not change at the level of overall abundance after Aβ induction (Fig. S2A) [51]. In parallel, we sourced aging mRNA expression data from the “metaworm” dataset which includes 60 aging transcriptomic profiles for wild-type animals [52]. We performed Spearman’s rank correlation analysis on mRNA expression changes of CIP proteins from day 2 to day 10, matching the age at which animals were collected for insoluble proteomics. Most CIP proteins did not significantly correlate with aging and, of those that did, there was an equal proportion of genes that increased or decreased (Fig. S2A&B). Finally, using publicly available lysate proteomics data for aging wild-type *C. elegans*, we found that the overall abundance of 94% of CIP proteins did not significantly change during aging (Fig. S2A) [53]. Collectively, these data strongly refute the notion that expression changes are a primary driver of CIP protein insolubility.

Intriguingly, while the aging-driven insoluble proteome was indistinguishable from the CIP, differences emerged between the CIP and the Aβ-driven insoluble proteome (Fig. S2B-E). Specifically, the Aβ-driven insoluble proteome displays greater aggregation propensity and lower solubility than the CIP (Fig. S2B & C) [49]. These data are consistent with the notion that Aβ and aging drive protein insolubility through a common mechanism but that Aβ may also cause insolubility through a distinct mechanism, likely by direct interaction and seeding of aggregation. Together these findings support the hypothesis that aging and Aβ act synergistically in the context of AD to accelerate age-related loss of proteostasis.

### Protein insolubility impacts biological processes that increase human age-related disease risk

**Fig. 3:**
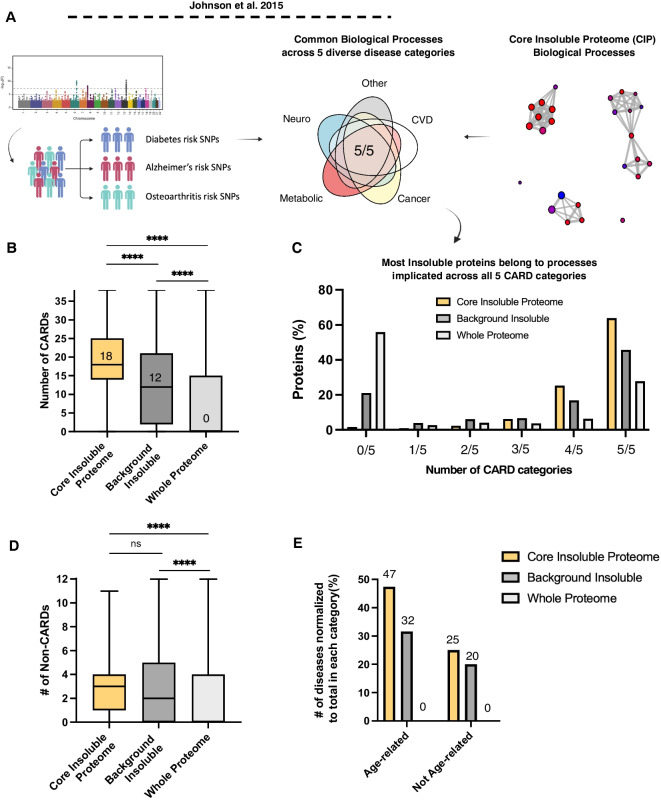
The insoluble proteome is enriched with modifiers of age-related disease risk. The core insoluble proteome is enriched with modulators of age-related disease risk. **A** Schematic representation of GWAS data53 used to compare GO biological processes (BP) shared across chronic, age-related diseases (CARDs) with the BPs represented in the CIP, created with Biorender.com. **B** Distribution of the number of CARDs that share biological processes with proteins found in CIP, the background insoluble proteome, and the *C. elegans* reference proteome with the median value displayed, Kruskal–Wallis test with Dunn’s, error bars = range. **C** Frequency distribution of the number of broad CARD classes that share one or more biological processes with proteins found in the CIP, the insoluble proteome, and the proteome. **D** Distribution of number of non-age-related diseases, Kruskal–Wallis test with Dunn’s, error bars = range. **E** Proportion of diseases sharing annotations with the insoluble proteome normalized to the number of diseases tested in either age-related disease or non-age-related disease categories

KEGG analysis of the CIP showed that in addition to bearing annotations for neurodegenerative disease pathways, many insoluble proteins bore pathway annotations for non-neurological, age-related diseases, such as non-alcoholic fatty liver disease and diabetic cardiomyopathy (Supp. Tables 9A & B). This led us to question if the relationship between the insoluble proteome and chronic age-related disease (CARD) could go beyond simply neurodegeneration. We reasoned that biological pathway dysfunction due to insolubility could be an underappreciated driver of CARD risk. Previous GWAS analyses demonstrated that common biological processes underly a whole array of diverse CARDs [54]. We therefore applied this analytical pipeline to query the relevance of protein insolubility and biological processes implicated in CARD risk (Fig. 3A). Specifically, we compared biological processes impacted by insolubility against those implicated in the risk of developing 38 distinct CARDs (Supp. Table 10). We found that, on average, proteins in the CIP shared biological processes with 18 of the 38 different CARDs (Fig. 3B). Moreover, 88% of the CIP proteins shared annotations with diseases spanning four or more of five broad CARD categories: neurodegenerative, metabolic, cancer, cardiovascular, and “other” (capturing disparate CARDs such as macular degeneration, rheumatoid arthritis, and osteoporosis) (Fig. 3C). This association was enriched compared with the experimental background insoluble proteome (i.e., all proteins that could be identified in the insoluble fraction across all experimental conditions) and highly enriched compared with proteins in the whole proteome for which the average protein had no shared processes with CARDs (Fig. 3B).

We questioned if this enrichment was specific for age-related diseases or simply an association with diseases more generally. We therefore repeated the analysis with 12 non-age-related diseases such as schizophrenia, childhood leukemia, and autism. We found that the CIP shared biological processes with only 3 diseases and there was no statistical difference between the CIP and the experimental background insoluble proteome (Fig. 3D). Normalizing by the number of disease comparisons showed a roughly twofold higher enrichment for CARDs over non-age-related diseases (Fig. 3E). Taken together these data suggest that the CIP is enriched with processes whose dysfunction contributes to disease risk but age-related disease risk in particular.

It is worth noting that TOMM40, which we identified in the Aβ-driven insoluble proteome and two independent aging-driven insoluble proteome datasets [10, 28], is one of only a very small fraction of SNPs (2.5%) that increase disease risk for > 3 broad categories of CARD suggesting that TOMM40 insolubility, and mitochondrial protein import deficits, could be central drivers of age-related disease [54].

The biological processes common to the insoluble proteome and all 5 broad CARD categories included: immune activation and stress response pathways; growth signaling, such as fibroblast growth factor (FGF) and WNT signaling; RNA splicing and regulation of expression; and tissue homeostatic processes important for development and wound healing, such as ECM organization, cell migration and cell differentiation (Fig. S3A). Except for a few rare cases, soluble proteins lose their biological function when they become insoluble, and therefore, these data provide suggestive evidence that insolubility of a core set of vulnerable proteins might promote risk of not only neurodegenerative disease but CARDs more broadly.

### The CIP can be used to identify therapeutic targets for Aβ toxicity

**Fig. 4:**
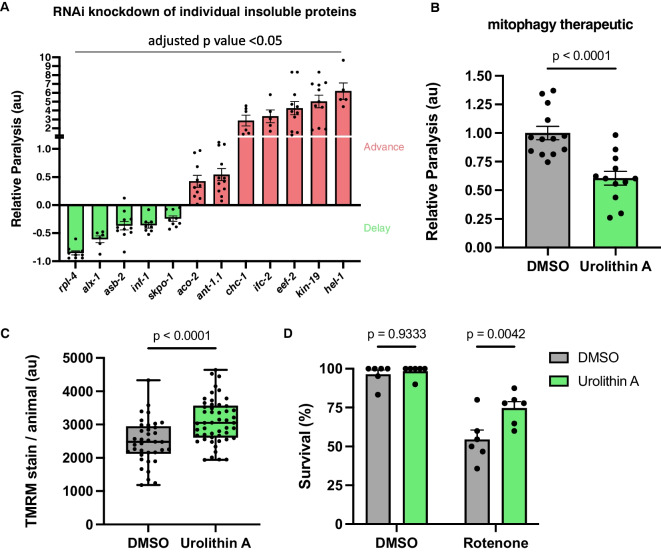
Targeting the insoluble proteome genetically or pharmacologically modulates Aβ toxicity. **A** Statistically significant hits from RNAi paralysis screen against CIP proteins. Each point represents the relative proportion of paralyzed animals on that plate normalized to the average paralysis on the control vector for that experiment, approx. 40 animals per plate, three separate experiments, error bars = SEM, for statistical tests see Supplementary Table 11A. **B** Barplot of paralysis under 50 µM Urolithin A treatment, 40 animals per plate, three experiment replicates, error bars = SEM, unpaired *t* test. **C** Boxplot of tetramethylrhodamine, methyl ester (TMRM), mitochondrial membrane potential stain in Aβ expressing animals normalized to body area after pre-treatment with 50 μM Urolithin A, 40–50 animals per condition, bars = min. to max., unpaired *t* test. **D** Barplot of survival of Aβ expressing animals in 50 µM rotenone solution after pre-treatment with 50 µM Urolithin A, approx. 75 animals per condition, error bars = SEM, two-way ANOVA with Šídák’s multiple comparisons test

Given that one in six of the CIP proteins has been shown to alleviate toxicity in either HD or PD models, we speculated that insoluble proteins in the CIP could be playing a direct role in the toxicity of Aβ, and therefore reducing their expression may be beneficial. To test this, we knocked down the expression of a subset of CIP-encoding genes using RNAi in the same *C. elegans* Aβ model and measured paralysis. In parallel, we performed the experiment with non-transgenic animals to rule out any non-specific effects on muscle function. We found that, of the 23 genes tested, 12 significantly impacted paralysis, eight had no impact, and three were either lethal or significantly delayed development and so could not be quantified (Fig. 4A). Therefore, of the CIP genes we were able to test, 60% significantly impacted Aβ toxicity (Fig. 4A Supp Tables 11A & B). Contrary to our initial hypothesis, however, most of these genes (7/12) exacerbated paralysis rather than preventing it. This may be because knocking down mRNA expression of some proteins could be further reducing the levels of the soluble protein thereby compounding loss of function due to insolubility. When compared against a knockdown screen of ~ 8000 protein-coding genes in a similar Aβ *C. elegans* model, our results represent a roughly 60-fold greater hit rate, suggesting that the CIP is highly enriched with disease-modifying proteins [55].

Since we observed strong evidence of mitochondrial protein unfolding in the insoluble proteome, we hypothesized that the CIP could be targeted by pharmacologically enhancing mitophagy with a small molecule. Urolithin A (UA) is a mitophagy-inducing, natural product derived from the bacterial metabolism of foods containing ellagitannins, such as pomegranate seeds and walnuts, in the gastrointestinal tract [56–58]. UA increases lifespan and protects neurons from amyloid toxicity in *C. elegans* through a mitophagy-dependent mechanism [59, 60]. UA supplementation in older adult humans was shown to improve muscle function in a phase II clinical trial for age-related frailty [61, 62]. We treated Aβ-expressing animals with UA for 18 h prior to inducing Aβ expression and scored paralysis after 24 h. We measured a robust and significant decrease in paralysis (Fig. 4B, Supp. Table 12). To confirm that UA improved mitochondrial quality we assayed mitochondrial function in Aβ expressing animals. Previous work demonstrated that Aβ expression caused a reduction in mitochondrial membrane potential [63], which we found to be significantly rescued by UA treatment (Fig. 4C). Additionally, we found that UA increased resistance to the mitochondrial complex I-specific inhibitor, rotenone (Fig. 4D). Taken with recent Aβ mouse model data [64], these results support that targeting mitochondrial quality control by mitophagy represents a reasonable strategy for preventing or treating Aβ toxicity.

## Discussion

In this study, we used an unbiased proteomics approach to assess the impact of Aβ on protein insolubility. We discovered that Aβ expression is sufficient to cause proteome-wide protein insolubility and in particular unfolding of the mitochondrial ETC and membrane transporters. We found that the Aβ-driven insoluble proteome is highly similar to the aging-driven insoluble proteome, suggesting there exists a core sub-proteome, vulnerable to insolubility under stress conditions. This core insoluble proteome was remarkably enriched for regulators of lifespan and disease pathology in *C. elegans*; with a particularly strong enrichment for regulators of neurodegenerative proteinopathies. Using analysis of GWAS data [54] we uncovered suggestive evidence that protein insolubility could link diverse CARDs by the disruption of common biological processes. This finding is particularly compelling considering the geroscience hypothesis, which “posits that aging physiology plays a major role in many — if not all — chronic diseases” [65]. While purely correlative at this stage, it leads us to hypothesize that insolubility could be a contributing factor to the etiology of CARDs more generally.

While this study focused on the impact of Aβ on protein insolubility we might expect that other aggregation-prone proteins that disrupt protein folding mechanism such as polyglutamine repeat proteins or α-synuclein could have a similar impact on overall protein solubility. It will be important to determine the specificity of the insoluble proteome phenotype for disease-associated proteins versus simply aggregating proteins. For instance, expression of an aggregation-prone GFP in *C. elegans* led to a similar paralysis phenotype to Aβ expression but it is unknown if this correlates with proteome-wide protein insolubility. However, the pathogenic mechanism leading to paralysis in each model is likely different. Specifically, in the GFP strain toxicity is rescued completely by HSP-16 over-expression [66]; whereas multiple groups have shown that expression of HSP-16 class genes increases up to 80-fold in response to Aβ expression without any rescue [51, 67].

Expression of a polyglutamine repeat protein in *C. elegans* drives aggregation of endogenous, metastable proteins, such as Paramyosin, via sequestration of the proteostasis machinery [68]. Paramyosin increased 8.5-fold in the insoluble fraction upon Aβ expression, and it is quite possible that such a mechanism plays a role in Aβ-driven proteome-wide protein insolubility. Previous work demonstrated that the concentrations of intracellular proteins are close to their solubility limit and that during aging proteins lose solubility without changing at the level of expression [50]. The authors propose that changes in the intracellular environment, such as declining chaperone function, could decrease the effective solubility limit of proteins thereby leading to insolubility [50]. The fact the CIP is enriched with supersaturated proteins lacking intrinsic aggregation propensity in the absence of expression changes is consistent with a model in which intracellular proteostasis changes lead to a reduction in effective solubility and proteome-wide insolubility.

The Aβ-driven insoluble proteome data is consistent with evidence in yeast and human cells that Aβ, and specifically the Aβ_1-42_ peptide, interferes with the import of mitochondrial protein precursors into the mitochondria by directly interacting with the TOM proteins [69–72]. We observed that the insoluble proteome is highly enriched with nuclear-encoded but not mitochondrial-encoded ETC complex proteins. Our data extend previous findings by providing the first evidence that outer membrane proteins themselves become insoluble due to Aβ, including GOP-3 (SAMM50) of the SAM complex which has not been implicated before. Further, we found that targeting mitochondrial protein homeostasis with an established mitophagy-inducing compound, Urolithin A, was efficacious in preventing Aβ toxicity which may explain its observed therapeutic effects in Aβ transgenic mice [64]. Interestingly, while many interventions that increase lifespan in *C. elegans* are associated with reduced fertility, UA was shown to increase reproductive span by increasing mitochondrial quality in the oocyte, further supporting its potential as a gerotherapeutic [73]. These data clearly support the notion that Aβ disrupts mitochondrial function through loss of mitochondrial protein homeostasis, and possibly via aggregation of mitochondrial outer membrane protein transporters, warranting further attention.

Here, we demonstrated that insoluble proteomics can be used to identify modulators of Aβ toxicity. We show that reducing the expression of specific proteins in the insoluble proteome can either advance or delay Aβ toxicity in *C. elegans*. We observed counter-intuitive effects which could point towards specific disease-modifying mechanisms for future research. Firstly, knockdown of the large ribosomal protein RPL4 caused a robust suppression of paralysis. This was anticipated because we assumed this would reduce global translation and thus reduce the overall demand on the proteostasis machinery. However, knockdown of the translation elongation factor EEF-2, which should have similarly decreased global translation, had the opposite effect, resulting in a profound increase in paralysis. Similarly, we also observed a counter-intuitive result when targeting insoluble mitochondrial proteins. Knocking down the mitochondrial ATP synthase peripheral stalk protein, ASB-2, for example, was found to significantly delay paralysis. In contrast, knocking down the mitochondrial membrane transporter ANT-1.1 or the aconitase enzyme ACO-2 led to significantly worse paralysis. RNAi against ASB-2 and other subunits of the ATP synthase complex are known to increase lifespan [74, 75], possibly through a mito-hormetic response, and this could explain the protective effects observed in our model. However, ANT-1.1 and ACO-2 knockdown have also been shown to increase lifespan by several independent groups [4, 75–78], demonstrating a more complex picture. While these data could point to a compensation response, we speculate that certain insoluble proteins might serve a cytoprotective role in sequestering misfolded proteins or, in this case, toxic Aβ monomers, into inert, insoluble aggregates. Knockdown of other members of the insoluble proteome might initiate a protective response at the level of transcription or protein homeostasis machinery.

Over 50 biological processes with known links with age-related disease risk were shown here to be linked with insolubility during normal aging (Fig S3A). These data suggest that clinicians and scientists working on CARD mechanisms could consider the role of protein insolubility when studying the interplay between aging and their disease of interest; particularly of biological processes implicated in this study. We propose that evidence exists to support this approach when thinking about therapeutic strategies for age-related diseases. For example, the small molecule, HBX, which slows aging by increasing protein homeostasis in invertebrate models had unexpected, positive effects on bone aging in the laboratory mouse [79]. Based on the discovery of a core insoluble sub-proteome and the processes implicated in CARD risk, targeting the insoluble proteome could provide an encompassing strategy for the prevention and treatment of disparate age-related diseases.

## Experimental methods

### Animal strains

The following strains were used to generate the proteomics data: GMC101 (dvIs100 [*unc-54*p::A-beta-1–42::*unc-54* 3'-UTR + *mtl-2p*::GFP]), CL2122 (dvIs15 [(pPD30.38) *unc-54*(vector) + (pCL26) *mtl-2*::GFP]). GMC101 was used to test Urolithin A for protection against paralysis.

The following *C. elegans* strain was generated for this study, GL399 (dvIs100 [*unc-54*p::A-beta-1–42::*unc-54* 3'-UTR; *spe-9*(hc88) I; *rrf-3*(b26) II.), by crossing TJ1060(*spe-9*(hc88) I; *rrf-3*(b26) II) with GMC101 (dvIs100 [*unc-54p*::A-beta-1–42::*unc-54* 3'-UTR + *mtl-2p*::GFP]). GL399 was used for all RNAi paralysis assays.

### Animal maintenance

GMC101 and CL2122 animals were maintained at 20 °C on 60 mm Nematode Growth Media (NGM) agar plates seeded with OP50 *E. coli*. Animals were maintained by transferring 30–50 eggs to a fresh plate on Monday and Friday. GL399 was maintained at 15 °C on 60 mm NGM agar plates seeded with OP50 *E. coli*. Animals were maintained by transferring 30–50 eggs to a fresh plate weekly.

### Insoluble protein extraction

Isolation of SDS-insoluble proteins from worms was completed as described in detail in [28]. Briefly, 200 day 2 adult animals were allowed to lay eggs for 5 h on 100 mm NGM agar plates seeded with 4 × concentrated OP50 *E.coli*. After 50–52 h animals were collected in sterile S-basal solution (5.85 g NaCl, 1 g K_2_ HPO_4_, 6 g KH_2_PO_4_, 1 ml cholesterol (5 mg/ml in ethanol) per 1 L sterile H_2_O) and transferred to fresh 4 × OP50 seeded plates. At 72 h post egglay animal plates were transferred to 25 °C to initiate Aβ expression. Twenty-four hours later, animals were collected in sterile S-basal. The animals were allowed to settle under gravity and the supernatant solution was removed. This step ensured larval worms would not be taken forward for insoluble protein isolation. The adults were washed several times to remove all bacteria and larvae and then the supernatant was removed and the tubes flash-frozen in dry ice/ethanol.

Animal pellets were thawed in the presence of detergent-free lysis buffer containing protease inhibitor (20 mM Tris base, pH 7.4, 100 mM NaCl, 1 mM MgCl_2_). Thawed pellets were then sonicated in a water bath kept at 4 °C on maximum intensity for 30 s ON and 30 s OFF to prevent sample overheating. After the first round of sonication for 10 cycles (10 min) pellets were checked under the light microscope to ensure animals were efficiently lysed. Lysates were clarified by centrifugation at 3000 g for 4 min. Supernatant was collected, and total protein was quantified by BCA.

Insoluble proteins were extracted by aliquoting lysate equivalent to 2 mg of total protein and spinning at 20,000 g for 15 min at 4 °C. The supernatant was collected as the aqueous-soluble fraction. The insoluble protein pellet was resolubilized in 1%(w/v) SDS lysis buffer (20 mM Tris base, pH 7.4, 100 mM NaCl, 1 mM MgCl_2_ plus protease inhibitor) and centrifuged at 20,000 g for 15 min at room temperature. The supernatant was collected as the 1% SDS-soluble fraction. The pellet was then resolubilized and washed twice more in 1% SDS lysis buffer, and the supernatant fractions were kept. The remaining SDS-insoluble pellet was resolubilized in sterile 70% (v/v) formic acid and sonication in a water bath for 30 min. Samples were then dried in a vacuum concentrator for 1 h to completely remove formic acid. 1 × LDS sample gel buffer was added to the dried pellet and the samples were heated to 95 °C for 10 min. Samples were briefly centrifuged to collect condensation and stored at − 80 °C or run onto a 4–12% NUPAGE Bis–Tris gel.

### Mass spectrometric acquisitions

Dried pellets were dissolved in 40 µL of 1 × LDS sample gel buffer, incubated at 95 °C for 10 min, vortexed, and spun down. Solubilized samples were run in pre-cast NuPAGE 4–12% gradient acrylamide Bis–Tris protein gels (Invitrogen) for 20 min to concentrate the proteins in a single band in the stacking gel. For in-gel digestion, the gel bands were diced, collected in tubes, and dehydrated with a dehydration buffer (25 mM ammonium bicarbonate in 50% acetonitrile (ACN) and water). The gel samples were dried in a vacuum concentrator, reduced with 10 mM dithiothreitol (DTT) in 25 mM ammonium bicarbonate (pH 7–8) and incubated for 1 h at 56 °C with agitation, then alkylated with 55 mM iodoacetamide (IAA) in 25 mM ammonium bicarbonate (pH 7–8), and incubated for 45 min at room temperature in the dark. The diced gel pieces were washed with 25 mM ammonium bicarbonate in water (pH 7–8), dehydrated again with the dehydration buffer, and dried in a vacuum concentrator. Afterwards, 250 ng of sequencing-grade trypsin in 25 mM ammonium bicarbonate (pH 7–8) was added to each sample. Gel pieces were vortexed for 10 min, briefly spun, and incubated at 4 °C for 30 min without mixing. Gel pieces were covered with 25 mM ammonium bicarbonate (pH 7–8) and incubated overnight for 16–20 h at 37 °C with agitation. Subsequently, the digested peptides were further extracted, as gel pieces were subjected to water and then two separate additions of a solution containing 50% ACN, 5% formic acid (FA) in water. After each addition of solution, the sample was mixed for 10 min, and then the aqueous digests from each sample were transferred into a new tube. These pooled peptide extractions were dried in a vacuum concentrator until completely dry. Proteolytic peptides were re-suspended in 30 µL of 0.2% FA in water and desalted using stage tips made in-house containing a C_18_ disk, concentrated in a vacuum concentrator, and re-suspended in 15 µL of 0.2% FA in water and 1 µL of indexed Retention Time Standard (iRT, Biognosys, Schlieren, Switzerland).

Samples were then subjected to mass spectrometric analysis using a high-performance liquid chromatography (HPLC) system combined with a chip-based HPLC system (Eksigent nano-LC) directly connected to a quadrupole time-of-flight mass spectrometer (TripleTOF 5600, a QqTOF instrument) as detailed in our previous step-by-step protocol [28].

### DIA/SWATH data processing and statistical analysis

Each sample was acquired in data-dependent acquisition (DDA) mode to build peptide spectral libraries, as we previously described [28]. Data-Independent Acquisition (DIA)/SWATH data was processed in Spectronaut (version 12.0.20491.3.15243) using DIA. Data extraction parameters were set as dynamic and non-linear iRT calibration with precision iRT was selected. DIA data was matched against an in-house *Caenorhabditis elegans* spectral library that provides quantitative DIA assays for 5461 control and 10,140 GMC 101 *C. elegans* peptides corresponding to 1223 protein groups for control and 1839 protein groups for GMC 101 and supplemented with scrambled decoys (library size fraction of 0.01), using dynamic mass tolerances and dynamic extraction windows. DIA/SWATH data was processed for relative quantification comparing peptide peak areas from different days. Identification was performed using 1% precursor and protein *q*-value. Quantification was based on the peak areas of extracted ion chromatograms (XICs) of 3 – 6 MS2 fragment ions, specifically b- and y-ions, with *q*-value sparse data filtering and iRT profiling being applied. For this sample set, local normalization was not implemented. Differential protein expression analyses for all comparisons were performed using a paired *t*-test, and *p*-values were corrected for multiple testing, using the Storey method. Specifically, group-wise testing corrections were applied to obtain *q*-values. Protein groups with at least two unique peptides, *q*-value < 0.01, and absolute Log_2_(fold-change) > 0.58 are significantly altered (Supp. Tables 1,11A,11B).

### Data accession

Raw data and complete MS data sets have been uploaded to the Mass Spectrometry Interactive Virtual Environment (MassIVE) repository, developed by the Center for Computational Mass Spectrometry at the University of California San Diego, and can be downloaded using the following link: https://massive.ucsd.edu/ProteoSAFe/private-dataset.jsp?task=d7c68fbc04ce4f5e9c757cd14daa7585 (MassIVE ID number: MSV000092250; ProteomeXchange ID: PXD043250). Enter the username and password in the upper right corner of the page: Username: MSV000092250_reviewer; Password: winter.

#### Computational methods

Enrichment analysis.

All enrichment analyses were performed using significantly increased proteins (Log_2_(fold-change) > 0.58, Q < 0.01) and gProfiler [80] with Benjamini-Hochberg < 5% FDR correction. Enrichment results are presented as the product of gene count and FDR-corrected *p*-value. Human orthologs were derived from Ortholist [32].

Protein biophysical predictions.

For intrinsic aggregation propensity we used the intrinsic solubility prediction we used the CamSol solubility online tool [47]. For LLPS propensity we used the catGRANULE score online tool [48]. For Zyggregator and supersaturation predictors, we sourced values from Ciryam et al. [49].

mRNA expression analysis.

Pre-normalized mRNA expression values were sourced from “metaworm” [52] for analysis of aging expression changes, across different laboratories for N2 wild-type *C. elegans*. Data was filtered to include only expression data from day 2 to day 10 of adulthood. To find direction of expression changes from day 2 to day 10, we performed Spearman’s Correlation analysis with significance cut-off p.adj < 0.05.

GWAS analysis.

All SNP GO biological process data for the present study was taken from Johnson et al. [54]. All GO biological process data for insoluble proteins was downloaded from DAVID [81, 82] and Uniprot [83]. For comparison between GO biological processes enriched in CARD risk with those represented in the insoluble proteome and CIP (the overlap between the Aβ-driven and aging-driven insoluble proteome), we determined overlaps between GO term annotations for the proteins and the CARDs. We used these overlaps then to determine the shared biological processes in the CIP and insoluble proteome with the five broad age-related disease categories as defined in Johnson et al. [54]. The “background insoluble” proteome was defined as any protein which could be reliably identified in the insoluble fraction in either of the experiments, i.e., the experimental background. For comparison with the “whole proteome” writ large, we generated a random list of 1600 proteins from the *C. elegans* proteome using a random number generator and downloaded their GO biological process data from DAVID and Uniprot. GO biological processes shared between the CIP and all five diverse categories of CARD were processed through ReviGO [84] to remove any redundant terms and create a relationship network between the processes, which we subsequently modified in Cytoscape [85] and presented in Fig. S3.

#### Urolithin A paralysis assay

A 20 mM stock of Urolithin A (UA) was prepared in sterile DMSO and stored in aliquots at − 20 °C. From the stock solution, 130 µL of the working solution (50 µM) was prepared by mixing 7.5 µL of stock solution (or DMSO only for control plates) with 125.5 µL of distilled sterile water and was added to the top 35 mm NGM plates (3 mL NGM agar) pre-seeded with OP50 *E. coli*. A population of synchronized GMC101 animals was generated by allowing day 2 adults to lay eggs for 2 h at 20 °C on 60 mm NGM agar plates seeded with OP50. Progeny were allowed to develop to larval stage 4 (L4) and then transferred to NGM plates overlaid with 50 µM UA (or DMSO only for control plates, 0.05% DMSO). After 18 h, these plates were moved to 25 °C to initiate Aβ expression. Animals were scored for paralysis after a further 24 h. To score paralysis in an unbiased way, animals were moved to one quadrant of the plate and scored as paralyzed if they were unable to move away.

#### RNA interference paralysis assay

When the GMC101 strain is cultured on the RNAi HT115 bacteria, paralysis is significantly delayed. In addition, sterilization via 5-Fluoro-2′-deoxyuridine (FUDR) treatment completely protects from Aβ-induced paralysis. Therefore, to perform an RNAi paralysis screen in the absence of FUDR, it was necessary to generate a temperature-sensitive sterility mutant expressing Aβ. We therefore generated a new strain, GL399, by crossing the GMC101 Aβ expressing strain with TJ1060 which possesses a temperature-sensitive *Spe-9* mutation. Consequently, when GL399 is cultured at 25 °C, they become infertile at the same time as expressing Aβ. This allowed us to perform the paralysis assay over a longer time course. *Spe-9* has no impact on lifespan and did not prevent paralysis in response to Aβ expression. A population of synchronized GL399 worms was generated by allowing day 2 adults to lay eggs for 2 h at 20 °C on 60 mm NGM agar plates seeded with HT115 *E. coli* expressing the scrambled interfering RNA, or control vector RNAi. Progeny was allowed to develop for 48 h and then transferred to NGM plates seeded with HT115 *E. coli* expressing either the scrambled, control vector RNAi or RNAi against the gene of interest and immediately shifted to 25 °C to initiate Aβ expression. Animals were scored for paralysis after 50 h and 72 h to identify RNAi conditions that advance or delay paralysis, respectively. To score paralysis in an unbiased way, animals were moved to one quadrant of the plate and scored as paralyzed if they were unable to move away. Graphed results reflect the summarized findings of the two timepoints. Statistical tests performed and significance values are contained within Supplementary Tables.

#### Mitochondrial membrane potential, TMRM staining

TMRM staining was performed as previously described [86] but with modification to suit the GMC101 strain. TMRM plates were prepared by preparing a working solution from a 10 µM frozen stock and overlaying on pre-seeded OP50 plates containing DMSO or 50 µM UA, to give a final concentration of 150 nM TMRM. Plates were kept wrapped in foil at 20 °C for 24 h before use. Drug plate preparation was carried out as described for UA paralysis assay.

A population of synchronized GMC101 animals was generated by allowing day 2 adults to lay eggs for 2 h at 20 °C on 60 mm NGM agar plates seeded with OP50. At L4 stage, animals were moved to plates containing DMSO or 50 µM of UA. After 18 h, animals were moved to fresh plates containing DMSO or 50 µM of UA and 150 nM of TMRM and shifted to 25 °C. After 20 h, < 10% had paralyzed. Non-paralyzed animals were moved to fresh plates containing DMSO or 50 µM of UA without TMRM to de-stain for 1 h at 20 °C. After 1 h, 40–50 animals per condition were mounted on an agar pad on a glass microscope slide and immobilized using 5 mM levamisole. TMRM fluorescence was visualized with a Zeiss Imager Z1 fluorescence microscope using rhodamine filters with 2.5 s exposure for all samples. Quantification of images was performed using NIH ImageJ software. Fluorescent intensity of TMRM stain was measured per animal and normalized to body size.

#### Mitochondrial complex I inhibitor, rotenone, toxicity assay

Rotenone toxicity assay was performed as previously described [86] but with modification to suit the GMC101 strain. A population of synchronized GMC101 animals was generated by allowing day 2 adults to lay eggs for 2 h at 20 °C on 60 mm NGM agar plates seeded with OP50. At L4 stage, animals were moved to plates containing DMSO or 50 µM of UA. After 18 h, animals were moved to fresh plates containing DMSO or 50 µM of UA and moved to 25 °C. After 20 h, < 10% had paralyzed. Non-paralyzed animals were pooled from three to four agar plates into one plate before being transferred to the wells of a 96-well plate containing 50 µl of S-basal buffer with either DMSO or 50 µM rotenone. Animals were seeded at a density of 10–15 per well. To ensure dispersion of animals, the plate was shaken on a benchtop shaker for 15 s at 200 rpm. The 96-well plate was incubated at 20 °C for 3 h. Animals were then scored for survival and those that failed to display touch-provoked movement were scored as dead.

### Supplementary Information

Below is the link to the electronic supplementary material.

Supplementary file1 (PDF 1.29 MB)

Supplementary file2 (XLSX 2.02 MB)
