# Supplementary material for: Amyloid β accelerates age-related proteome-wide protein insolubility
Source: GeroScience. 2024 May 16;46(5):4585–602. doi: 10.1007/s11357-024-01169-1 (PMC11335993; doi:10.1007/s11357-024-01169-1)
Supplement: Supplementary file 1 — Supplementary file1 (PDF 1.29 MB) [file 11357_2024_1169_MOESM1_ESM.pdf]

Supplementary Figure 1

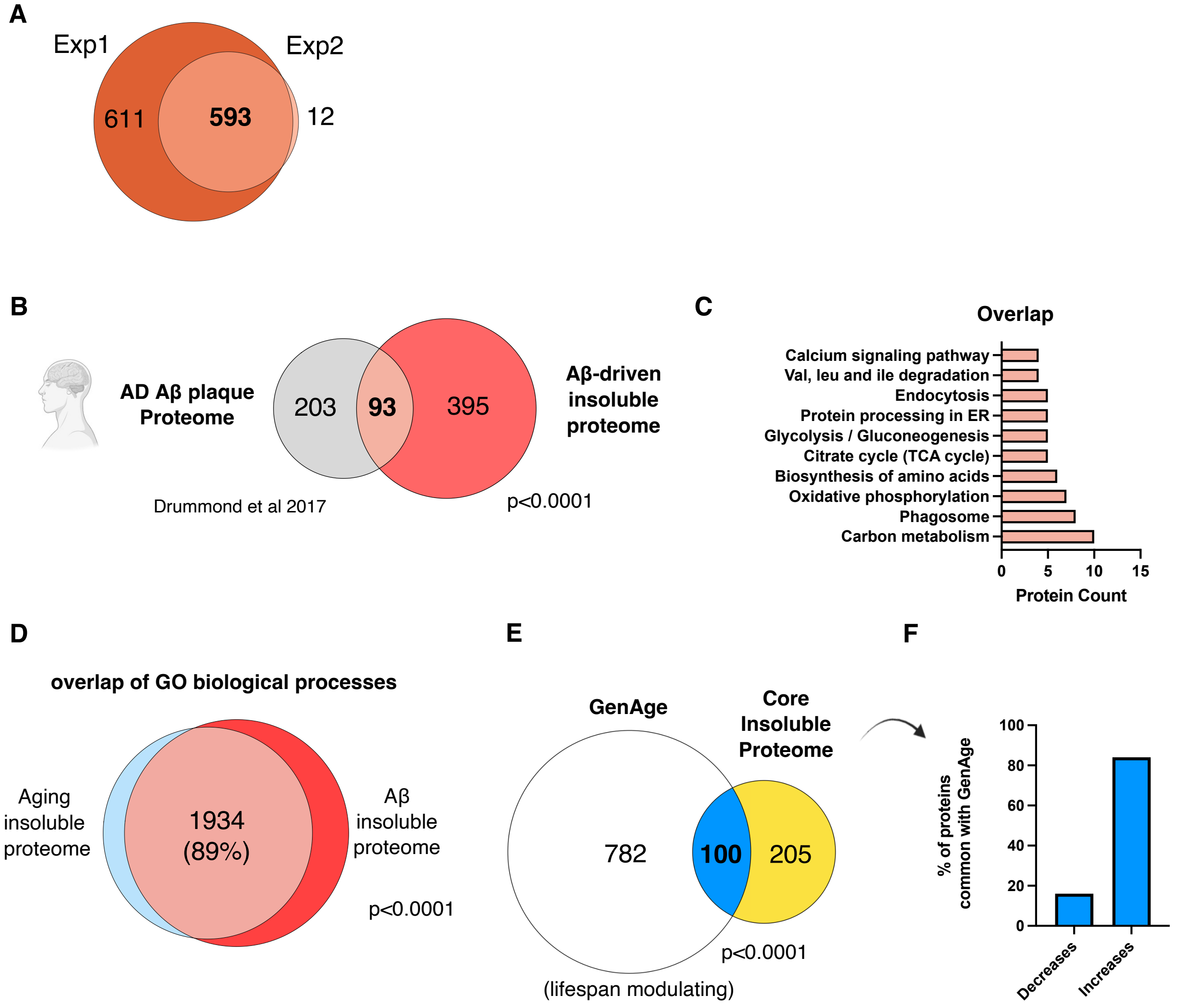

A. Overlap of proteins which increased in abundance in the insoluble proteome after Aβ expression across independent experiments. **B.** Overlap of proteins reliably identified in Aβ-rich plaques from AD patient brains[37] and the Aβ-driven insoluble proteome, Fischer's exact test, image created with Biorender.com. **C.** Top 10 KEGG annotations by protein count for overlapping proteins between AD senile plaque proteome (Drummond et al. 2017) and the Aβ-driven insoluble proteome. **D.** Overlap of GO biological processes represented in the aging insoluble proteome and the Aβ-driven insoluble proteome, Fischer's exact test. **E.** Overlap of proteins which have been shown to modulate lifespan (from GenAge database) and proteins in the Core Insoluble Proteome, Fischer's exact test. **F.** Proportion of proteins in the CIP for which reduced or ablated expression leads to a decrease or increase in lifespan in *C. elegans*.

Supplementary Figure 2

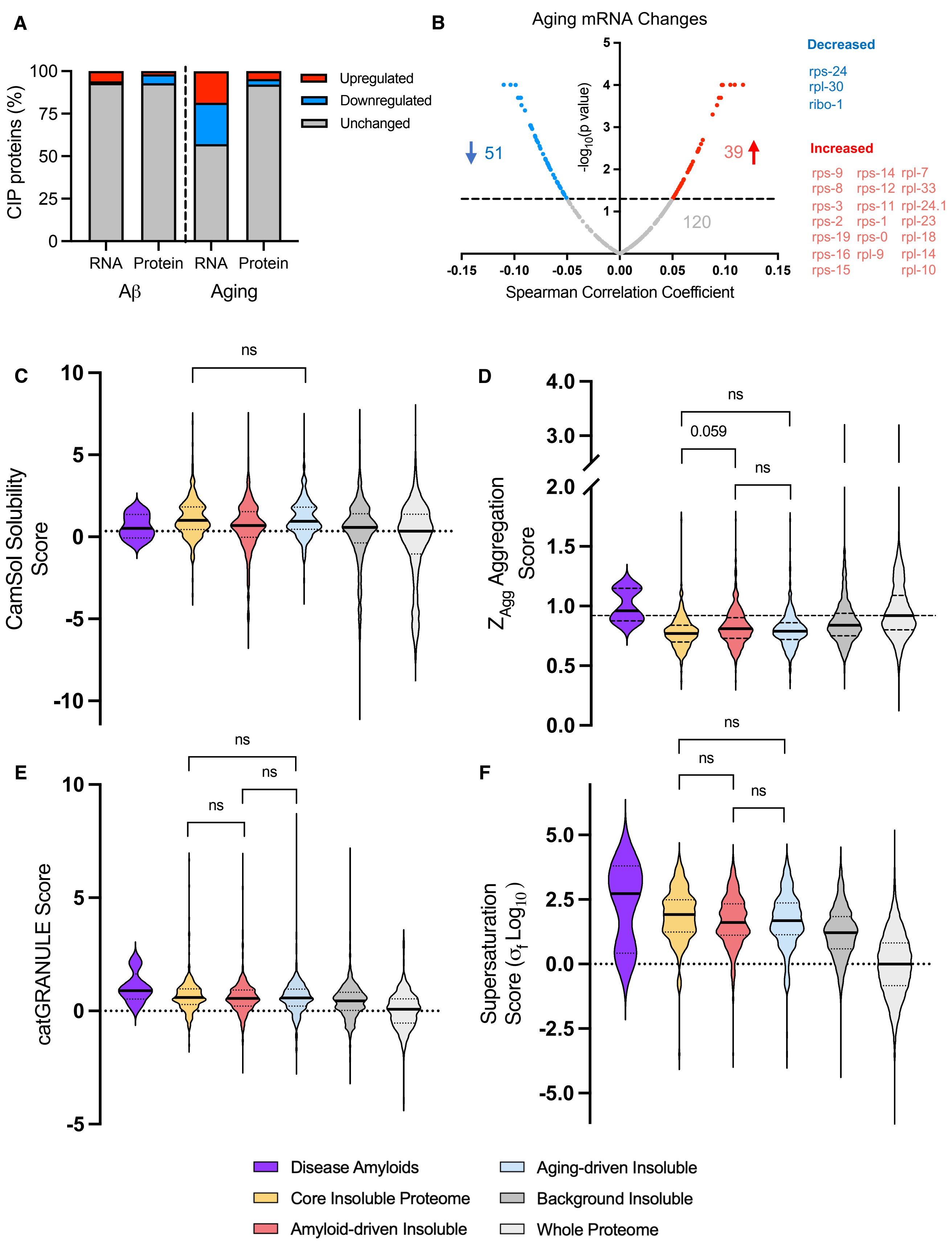

A. Proportion of CIP proteins differentially expressed after Aβ expression[50] or during wild-type aging in *C. elegans*[51,52]. **B** Volcano plot of Spearman Correlation between mRNA expression of CIP encoding genes and aging, from Day 2 to Day 10, in wild-type *C. elegans* with ribosomal genes highlighted. ***C.*** Violin plot of CamSol intrinsic solubility score distribution for each proteome. **D.** Violin plot of catGRANULE RNA granule prediction score distribution for each proteome. **E.** Violin plot of Zyggregator aggregation propensity score distribution for each proteome. **F.** Violin plot of Supersaturation ($\sigma_f Log_{10}$) score distributions for each proteome. In each case the Background Insoluble is all proteins identified in any insoluble proteome across any experiment and the Whole Proteome is the *C. elegans* reference proteome. Disease Amyloids = Aβ precursor protein, α-Synuclein, prion protein, β-2 microglobulin, and amylin. For clarity, only comparisons that were <u>not</u> statistically significant are shown, all other pair-wise comparisons were significant with p.adj<0.05, Kruskal Wallis Test with Dunn's correction.

Supplementary Figure 3

A **GO Biological Processes shared between the core insoluble proteome and all 5 ARD classes**

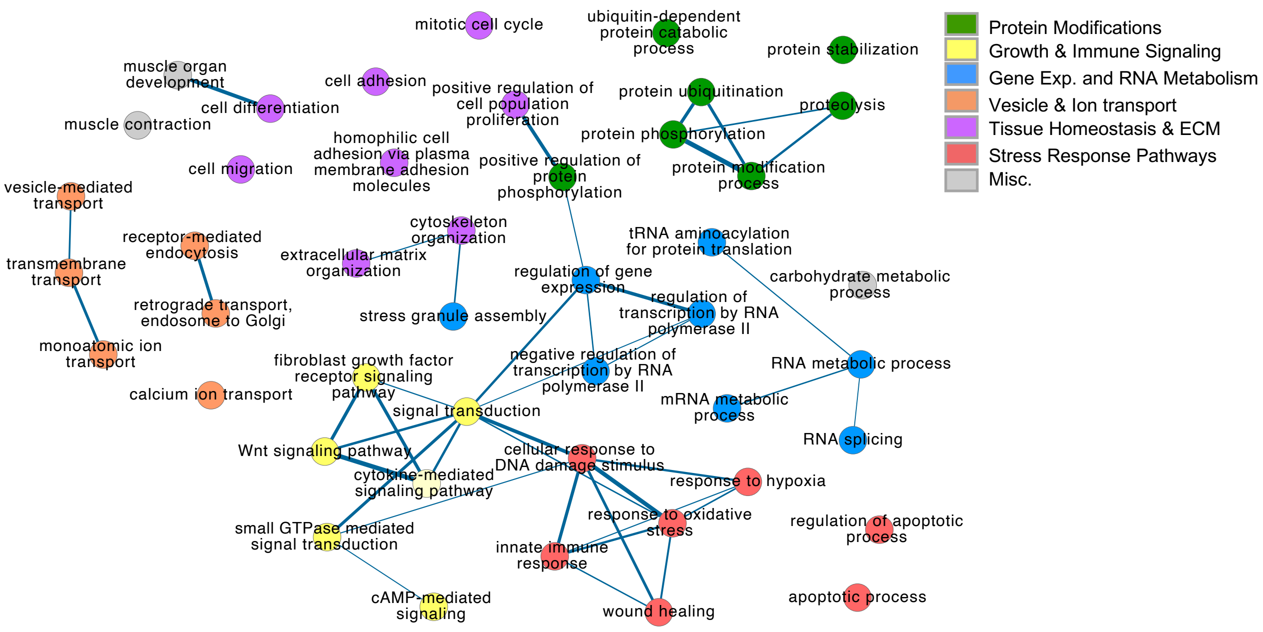

A. ReviGo network representation of the non-redundant GO biological processes shared between the CIP and all 5 diverse CARD categories.
